# Event-related functional Magnetic Resonance Spectroscopy

**DOI:** 10.1016/j.neuroimage.2023.120194

**Published:** 2023-05-26

**Authors:** Renée S Koolschijn, William T Clarke, I Betina Ip, Uzay E Emir, Helen C Barron

**Affiliations:** 1 Wellcome Centre for Integrative Neuroimaging, University of Oxford, FMRIB, John Radcliffe Hospital, Oxford, United Kingdom; 2 Donders Institute for Brain, Cognition and Behavior, Radboud University, Nijmegen, The Netherlands; 3 Medical Research Council Brain Network Dynamics Unit, University of Oxford, Oxford, United Kingdom; 4 School of Health Sciences, Purdue University, West Lafayette, United States

## Abstract

Proton-Magnetic Resonance Spectroscopy (MRS) is a non-invasive brain imaging technique used to measure the concentration of different neurochemicals. “Single-voxel” MRS data is typically acquired across several minutes, before individual transients are averaged through time to give a measurement of neurochemical concentrations. However, this approach is not sensitive to more rapid temporal dynamics of neurochemicals, including those that reflect functional changes in neural computation relevant to perception, cognition, motor control and ultimately behaviour. In this review we discuss recent advances in functional MRS (fMRS) that now allow us to obtain event-related measures of neurochemicals. Event-related fMRS involves presenting different experimental conditions as a series of trials that are intermixed. Critically, this approach allows spectra to be acquired at a time resolution in the order of seconds. Here we provide a comprehensive user guide for event-related task designs, choice of MRS sequence, analysis pipelines, and appropriate interpretation of event-related fMRS data. We raise various technical considerations by examining protocols used to quantify dynamic changes in GABA, the primary inhibitory neurotransmitter in the brain. Overall, we propose that although more data is needed, event-related fMRS can be used to measure dynamic changes in neurochemicals at a temporal resolution relevant to computations that support human cognition and behaviour.

## Introduction

Proton-Magnetic Resonance Spectroscopy (^1^H-MRS or MRS) is a non-invasive technique used to measure the absolute or relative concentration of neurochemicals. MRS takes advantage of the fact that the local chemical environment of protons (hydrogen nuclei) varies across different molecules. This leads to characteristic resonant frequencies of protons in different molecules, resulting in a distinct spectral profile for each neurochemical. A molecule can be detected with MRS if its concentration is sufficiently high, and its spectral profile is sufficiently non-overlapping with other chemicals. Typically, the spectra acquired using MRS are used to quantify the concentration of around 20 different neurochemicals. This includes glutamate and gamma-aminobutyric acid (GABA), the principal excitatory and inhibitory neurotransmitters. This quantification can be achieved using linear-combination modelling, an approach that uses the prior knowledge of known spectral patterns to model different neurochemicals, before translating model parameters into meaningful units of concentration.

By quantifying the concentration of a given molecule, MRS can be used to gain insight into neurochemical states in the brain, in both healthy and clinical populations (Öz et al., 2014). Depending on the experimental design (discussed in detail below), these neurochemical states may reflect homeostatic states, or be used to obtain more dynamic readouts of neurochemical concentration. However, a major challenge for MRS is that metabolites of interest, such as glutamate and GABA, are present at a low concentration compared to more abundant neurochemicals. Notably, the concentration of water in the brain is around 10,000 times higher than the concentration of neurochemicals of interest (Kreis, 2004). Therefore, signal from water molecules must be suppressed to prevent it from distorting the neurochemical spectrum.

The introduction of high field MR systems has benefited the detection of neurochemicals. The signal to noise ratio (SNR) of the MR signal scales with the main magnetic field strength (B_0_) of the MRI scanner. In addition, higher B_0_ field strength results in greater frequency separation of neurochemical signals in the spectrum. Consequently, moving from 1.5 T to 7 T enables separation of coupled spin systems between molecules such as glutamate and glutamine, and helps distinguish the spectral profiles for glutamate versus GABA (Pradhan et al., 2015; Tkáč et al., 2001).

The enhanced SNR associated with high and ultra-high B_0_ magnetic field strengths has also enabled the realization of functional MRS (fMRS), where MRS is coupled with a task or physiological intervention to provide a readout of the functional changes in neurochemical concentrations. Here we focus on *event-related* fMRS, a means to obtain *dynamic* readouts of neurochemicals that are thought to reflect functional changes in neural activity. Our review complements previous reviews that have discussed how blocked designs may be used to acquire fMRS data (Jelen et al., 2018; Koush et al., 2022; Mangia et al., 2009; Stanley & Raz, 2018). Here, we provide an in-depth review of *event-related fMRS*, building on previous work (Mullins, 2018) by including recent data acquired at higher field strength and discussing recent advances in software available for analysis. We also highlight important technical considerations and discuss appropriate interpretation of dynamic metabolic changes. Overall, we propose that event-related MRS can be used to capture dynamic, task-related changes in glutamate, GABA and other metabolites. We suggest such dynamic changes in neurochemicals have the potential to inform our understanding of human cognition and behaviour in a manner analogous to task-based functional Magnetic Resonance Imaging (fMRI).

## Task design for event-related MRS

**Figure 1:**
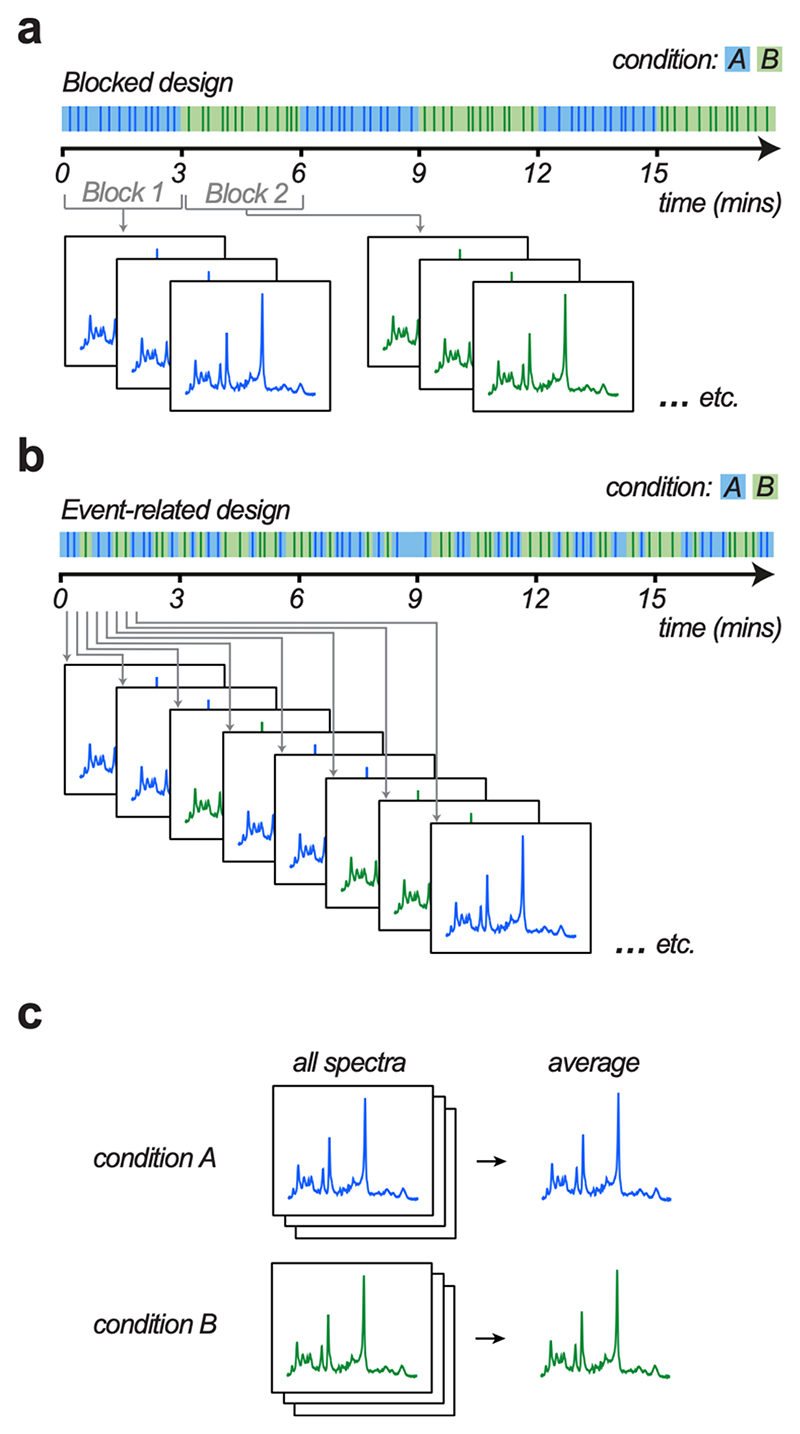
Blocked versus event-related functional MRS task design **a-b**. Schematic showing difference between ‘blocked’ and ‘event-related’ MRS task designs. Vertical lines represent trials. Shaded areas indicate time periods assigned to a particular condition. Two task conditions are included, *A* (blue) and *B* (green). In the ‘blocked’ design, multiple trials within each condition are presented consecutively, before there is a switch to the alternative condition. In the ‘event-related’ design, trials across the two conditions are presented in a random order. In both blocked and event-related designs, a jitter can be included between trials to optimise task design and minimise expectation effects. **c.** Schematic showing how MRS data acquired using both ‘blocked’ and ‘event-related’ can be analysed by taking all spectra from a given condition and applying processing to obtain an average spectra per condition (for example, (Koolschijn et al., 2021)). Alternatively, MRS data acquired using event-related design may be analysed using a General Linear Model based analysis applied to the time series (Apšvalka et al., 2015).

Both blocked and event-related experimental designs can be used to obtain time-resolved measures of neurochemicals with MRS. These two types of experimental design may be considered broadly analogous to those used for fMRI studies (Friston et al., 1999; Rosen et al., 1998). To date, the majority of MRS experiments have employed a blocked design, where experimental conditions within a task are divided up into blocks that typically span several minutes (Figure 1). As a point of comparison, we first give an overview of blocked fMRS designs, including their advantages and limitations, before discussing task design for event-related fMRS.

To analyse data from blocked fMRS studies, spectra within each block are averaged to give an estimate of the neurochemical concentration that can be mapped onto a particular experimental condition. Transition regions between blocks (i.e. the end of one block and the start of the next) may be excluded from further analysis. As observed in fMRI studies, blocked designs are efficient and they do not require an explicit model of the predicted neural response (Mechelli et al., 2003). At ultra-high field (7T), blocked designs using visual stimulation demonstrate consistent increases in the concentrations of neurochemicals such as glutamate and lactate in visual cortex for example, (Bednařík et al., 2015, 2018; Boillat et al., 2020; Fernandes et al., 2020; Ip et al., 2017; Lin et al., 2012; Mangia et al., 2007, 2009; Schaller et al., 2013), with similar results observed in motor cortex during motor stimulation (Schaller et al., 2014), which have been reproduced at lower field strengths (3T) when careful consideration is given to acquisition and analysis methodology (Volovyk & Tal, 2020). In addition to visual and motor stimulation, blocked designs at 4T have been used to show an increase in glutamate in the anterior cingulate cortex during painful stimulation (Mullins et al., 2005), and at 3T an increase in glutamate in the hippocampus during both encoding and retrieval of memory (Stanley et al., 2017). Blocked designs have also been used to effectively track changes in GABA with learning, demonstrating modulation in sensorimotor cortex during motor learning (Floyer-Lea et al., 2006) and in occipital cortex during a perceptual decision making tasks (Frangou et al., 2019). When applied to clinical populations, blocked designs show a reduced glutamatergic response in the anterior cingulate cortex in people with schizophrenia (Jelen et al., 2018; Taylor et al., 2015).

Overall, blocked designs can be used to show condition-specific equilibrium changes in neurochemicals which are thought to reflect stimulus specific increases in oxidative energy metabolism that accompany neuronal activation (Mangia et al., 2009). During blocks of visual stimulation (in this case at 8Hz), concurrent fMRI-fMRS in visual cortex reveals a correlation between the observed changes in glutamate and changes in the Blood-Oxygen Level Dependent (BOLD) signal (Ip et al., 2017). Similarly, increases in glutamate and lactate concentration can be observed during positive BOLD response, while decreases in glutamate and GABA can be observed during negative BOLD response (Boillat et al., 2020). However, when comparing responses in early visual cortex to perceived (7.5 Hz) and unperceived (30 Hz) changes in checkerboard stimulation, fMRS measures of glutamate and lactate, but not the BOLD signal, predict whether a stimulus is perceived (DiNuzzo et al., 2022). Depending on the experimental condition, task-induced changes in MRS may therefore occur in the absence of an equivalent neurovascular response, illustrating how fMRS blocked designs can provide unique information into neurochemical states relevant to cognition.

Functional changes in neurochemicals have also been demonstrated in rodents and other animal models using blocked designs. For example, blocks of visual stimulation in sedated mice reveals modulation of glutamate (Ligneul et al., 2021). Electrical stimulation applied to the trigeminal nerve is accompanied by an initial increase in lactate and glutamate in rat barrel cortex (Sonnay et al., 2017), while electrical paw stimulation gives significant increases in glutamate in the contralateral somatosensory cortex (Seuwen et al., 2019). Pre-clinical fMRS in sedated mice therefore appears sensitive to slow, progressive increases in glutamate that likely reflect the increase in oxidative metabolism that accompanies blocked stimulation (Bednařík et al., 2015). Importantly, unlike studies in humans, fMRS in rodents can be combined with stimulation protocols that include opto- or chemogenetic stimulation (Baslow et al., 2016; Just & Faber, 2019), thus providing an opportunity to test causality and elucidate the metabolic and neurotransmitter mechanisms that underlie fMRS-derived changes in neurochemicals. A key limitation of pre-clinical fMRS is that studies are typically performed in anaesthetized animals, while MRS in awake behaving animals presents its own challenges (Just, 2021). Moreover, the size of the rodent brain is small, making it highly difficult to obtain sufficient SNR from small regions of interest which are sensitive to partial volume effects. Ongoing technical refinement is therefore required to improve signal-to-noise through improved hardware, MR sequences and shimming techniques (Just, 2021).

For both human and pre-clinical studies, a significant limitation of fMRS blocked designs is the temporal resolution which is effectively set to the duration of each block. Blocked designs therefore obscure more rapid temporal dynamics of neurochemicals that underlie physiological processes relevant to ongoing cognition and behaviour. Furthermore, blocked designs cannot be used when task trials are classified post-hoc or are dependent on participant performance. Moreover, neurochemical concentrations appear susceptible to expectation effects where stimulus-induced changes in metabolite concentration decrease upon repeated presentation of the stimulus (Apšvalka et al., 2015; Mangia et al., 2007), analogous to repetition suppression effects reported with fMRI (Barron et al., 2016; Grill-Spector & Malach, 2001). Blocked designs are affected by these expectation effects, where task- or stimulus-induced increases in glutamate are reported to be larger or only present in the first experimental block (Bednařík et al., 2015; Taylor et al., 2015). Expectation effects must therefore be considered when assessing the relative sensitivity of a blocked design for detecting task-induced changes in neurochemicals using fMRS.

Event-related designs, on the other hand, involve presenting stimuli as a series of trials where different experimental conditions are intermixed across trials (Figure 1). Critically, this approach allows spectra to be acquired at a time resolution in the order of seconds. Event-related task designs control for expectation effects. In addition, trials can be categorised into different experimental conditions post-hoc, depending on the behavioural performance of the subject.

The SNR of a single MRS spectrum is typically too low to give a confident measure of neurochemical concentrations. Therefore, to quantify neurochemicals acquired using an event-related design, spectra may first be assigned to an experimental condition post hoc, before being averaged within condition to achieve sufficient SNR (Figure 1). As each spectra is acquired during a relatively fast event (stimulus presentation, behavioural response, inter-trial interval etc), averaging over instances of spectra that fall within the same condition still allows neurochemicals to be estimated at a temporal resolution of only a few seconds. Indeed, event-related fMRS has been used to reveal relatively large, transient changes in neurochemicals, as exemplified below. When locked to stimulus onset, these changes are reported to occur within 300-1000 ms (Apšvalka et al., 2015; Lally et al., 2014).

Rapid changes in neurochemicals may be described using a response function, analogous to the hemodynamic response function (HRF) reported using fMRI (Mullins, 2018). An estimate of the glutamate response function has been proposed using data from visual cortex acquired in two separate event-related studies. From these data sets, the peak is estimated at around 500 ms after stimulus onset and the response function returns to baseline 3–4 s after stimulus onset (Apšvalka et al., 2015; Lally et al., 2014; Mullins, 2018). However, to date there has been no formal study designed to map metabolite response functions. This leaves a critical knowledge gap that affects the reliability of estimating dynamic changes in event-related metabolite, discussed in more depth below (see *Analysing event-related fMRS data*). Thus, more work is needed to fully characterise metabolite response functions, together with any variation across brain regions. In addition, recent evidence suggests that neural decoding using event-related fMRI can be achieved at a sub-TR resolution, in the order of a few tens of milliseconds (Wittkuhn & Schuck, 2021). Using event-related MRS, it may similarly be possible to assess dynamic changes in neurochemicals at a sub-TR resolution. Overall, compared to blocked designs, where the lower limit of temporal resolution is set by the duration of the block (typically several minutes), event-related designs render a dramatic improvement in the temporal resolution of fMRS.

However, despite this apparent improvement in the temporal resolution, interpreting data acquired using event-related fMRS remains non-trivial. Notably, rapid changes in neurochemicals reported using event-related fMRS contrasts with findings reported in studies using blocked designs where more sluggish changes in the concentration of neurochemicals have been observed. To account for this slow response, previous studies using blocked designs have excluded up to 50% of data to obtain stable metabolite measurements (Bednařík et al., 2015; Mangia et al., 2006, 2007; Schaller et al., 2013), or the first two time averages of each block, equivalent to excluding 12.5% of the data (Ip et al., 2017). It remains unclear why this discrepancy in the timing of neurochemical changes is observed across event-related and blocked designs (see *Interpreting event-related fMRS data* below). One possibility is that event-related designs are more sensitive to changes in neurochemicals. Indeed, one meta-analysis of fMRS studies suggests that event-related fMRS designs measuring glutamate give a 2-3 times increase in effect size compared to blocked designs (Mullins, 2018). Another meta-analysis showed that a significant effect can be observed for glutamate across both event-related and blocked designs, while a significant effect for GABA is only observed for event-related designs (Pasanta et al., 2023). These findings should be interpreted with caution as in some cases only a few studies are considered, particularly meta-analyses assessing effects for GABA. Nevertheless, the reported increase in effect size for event-related fMRS studies may in part be explained by the fact that event-related designs mitigate against effects of expectation, habituation and adaptation (Apšvalka et al., 2015), factors that may reduce sensitivity to rapid changes in neurochemical dynamics when stimuli are repeated many times across a relatively long block.

A second possibility is that event-related and blocked designs are sensitive to different aspects of the glutamatergic and GABAergic response. The relatively slow changes in neurochemical concentration reported using blocked designs are thought to reflect changes in oxidative metabolism that occur with the increased energy demands of neural spiking activity (Mangia et al., 2009). More rapid changes in glutamate and GABA reported using event-related designs may instead reflect changes in the glutamatergic or GABAergic response that are more closely related to neurotransmission. However, as discussed in more depth below, MRS is generally considered insensitive to different pools of glutamate and GABA, with tight coupling between neurochemicals involved in metabolism and neurotransmission (Mangia et al., 2009). Furthermore, the concentration of glutamate and GABA in the synaptic cleft is too low to be detected using MRS. Thus, rather than indexing neurotransmission directly, event-related fMRS may detect changes in the visibility of glutamate and/or GABA to MRS that more closely reflect neurotransmission (Mullins, 2018). More data is needed, particularly in animal models, to support this working hypothesis and to establish the precise relationship between neurotransmitter release and MRS-derived neurochemical readouts.

**Figure 2:**
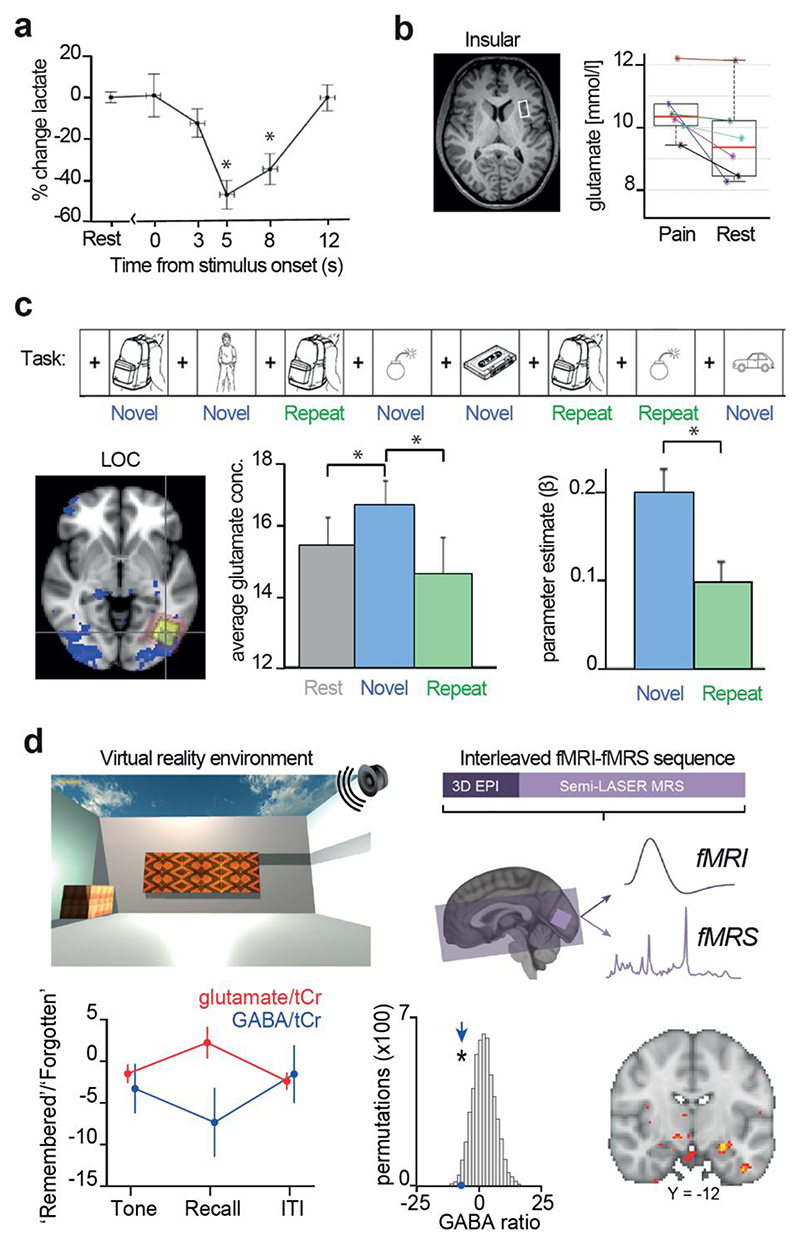
Data acquired using event-related fMRS **a.** Event-related fMRS used to detect changes in lactate in primary visual cortex in response to 1 s of visual stimulation with acquisition delay of 0 s, 3 s, 5 s, 8 s and 12 s. Plotted as a percentage with respect to rest (mean ± standard deviation). Adapted from (Mangia et al., 2003). **b.** Event-related fMRS used to detect increase in glutamate in anterior insular cortex in response to acute heat pain (‘Pain’) compared to rest (‘Rest’). Red: median; Upper and lower box plot limits show 75^th^ and 25^th^ percentiles, respectively. Adapted from (Gussew et al., 2010). **c.** Event-related fMRS used to demonstrate repetition suppression effects in glutamate. Upper: Schematic showing event-related fMRS task used to assess difference in neurochemical concentration between novel and repeated stimuli. Lower left: Mean (pink) and median (yellow) MRS voxel location, overlaid on significant BOLD response to stimuli (blue) in lateral occipital complex (LOC). Lower middle: Significant reduction in event-related glutamate response (mean ± 95% CI) to repeated stimuli compared to novel stimuli. Lower right: Significant reduction in event-related BOLD response to repeated stimuli compared to novel stimuli. *p < 0.05. Adapted from (Apšvalka et al., 2015). **d.** Event-related fMRS used to detect increase in glutamate:GABA ratio in primary visual cortex during recall of a visual cue. Upper left: Auditory-visual associative memory task performed in virtual reality. Upper right: schematic showing interleaved fMRI and fMRS sequence, with each data modality collected within each TR. Lower left: During recall of a visual cue the ratio between glutamate and GABA significantly increased in ‘remembered’ compared to ‘forgotten’ trials. Lower middle: Compared to a null distribution generated by permuting trial labels, the concentration of GABA significantly decreased during recall of a visual cue. Lower right: The increase in glutamate:GABA ratio during recall of a visual cue was predicted by the BOLD response in the hippocampus. Adapted from (Koolschijn et al., 2021).

With the above considerations in mind, studies implementing event-related MRS have investigated a wide range of cognitive processes. An early study by Mangia and colleagues used an event-related design to demonstrate a transient decrease in lactate in primary visual cortex about 5 seconds after onset of visual stimulation (Mangia et al., 2003) (Figure 2a). Notably, this finding contrasts with the increase in lactate reported during prolonged stimulation using a blocked design (e.g. (Bednařík et al., 2015)) and suggests that event-related designs are sensitive to lactate consumption that accompanies the onset of stimulus-locked neural activity. Another early use of event-related fMRS focused on changes in creatine/phosphocreatine and choline-containing compounds in response to emotional stimuli (Nishitani, 2003). The author found an increase in creatine/phosphocreatine and choline-containing compounds in hippocampus upon presentation of pleasant and unpleasant faces (Nishitani, 2003). As choline is thought to relate to the availability of acetylcholine (Lindner et al., 2017), a neuromodulator involved in attention and learning (Froemke & Martins, 2011; Hasselmo, 2006). A second study investigating choline by Lindner and colleagues (2017) found that choline in parieto-occipital cortex was modulated in accordance with shifts in attention (Lindner et al., 2017). Together these two studies demonstrate that event-related fMRS may be applied to study neurochemical changes that underlie cognitive processes such as attention.

Other groups have applied MRS to study the neural response to pain stimulation, where findings reported from studies using event-related designs (Cleve et al., 2015; Gussew et al., 2010) are generally consistent with those reported using blocked designs (Gutzeit et al., 2011, 2013; Mullins et al., 2005). For example, Gussew and colleagues (2010) found that glutamate concentration in insular cortex increased dramatically during painful heat stimulation (Gussew et al., 2010) (Figure 2b). Event-related changes in GABA+ have also been reported following heat stimulation (Cleve et al., 2015), where the authors applied a GABA-edited sequence (see section below entitled *Measuring event-related fMRS data*) by acquiring pairs of edited and non-edited spectra during consecutive stimuli, thus without sacrificing temporal resolution.

Finally, event-related fMRS paradigms have been used to target various cognitive processes involving visual areas, including in combination with other modalities like electro-encephalography (EEG) and BOLD fMRI. For example, Lally et al. (2014) combined fMRS in LOC with concurrent EEG, and found an increase in glutamate for object versus abstract visual stimuli, which in turn predicted the evoked gamma-band activity (Lally et al., 2014). Apsvalka et al. (2015) combined fMRS with BOLD fMRI to show a repetition suppression effect in glutamate for familiar stimuli in LOC (Apšvalka et al., 2015) (Figure 2c). A final example comes from the authors of this review, where an interleaved fMRI-fMRS sequence was applied to assess dynamic changes in glutamate and GABA in visual cortex (V1) during memory recall (Koolschijn et al., 2021) (Figure 2d). We found that memory recall of a visual stimulus was accompanied by a decrease in GABA and related to hippocampal BOLD.

Together, these studies demonstrate that event-related fMRS can be used to study a wide range of cognitive processes, ranging from pain to attention to associative memory, including in combination with other modalities like fMRI and EEG. However, detecting changes in neurochemicals like GABA, which are difficult to measure and quantify, remains non-trivial.

## Measuring event-related fMRS data

The concentration of neurochemicals is 10^4^ lower than the concentration of water in the brain. MRS therefore has much lower sensitivity than water-based MRI and it remains challenging to use event-related fMRS to measure neurochemicals that reside at relatively low concentration, have resonant peaks that overlap with more abundant neurochemicals, and/or have peaks with complex (multiplet) patterns resulting in low SNR. GABA, for example, suffers from all three issues, with one of its multiplet peaks overlapping with the total creatine singlet signals that are an order of magnitude larger. Increasing the B0 field strength increases SNR but also improves spectral resolution, which increases reliability of metabolite quantification (Mlynárik et al., 2008). Arguably, conventional MRS may only allow for reliable quantification of GABA at field strengths of 7T and above. Even at ultra-high field strengths (7T and above) excellent data quality and optimised analysis pipelines remain an imperative. At lower field strengths (i.e. 3T) reliable detection and quantification of event-related GABA may nevertheless be achieved by removing the strong overlapping resonances using J-difference spectral editing (Choi et al., 2021). In the following section we discuss the pros and cons of using spectral editing relative to non-edited sequences when acquiring event-related fMRS data, before discussing techniques available for improving the SNR of event-related fMRS.

The most common approach to measuring GABA involves using the MEscher-GArwood (MEGA) “J-difference” editing method (Puts & Edden, 2012). Here, for each measurement of GABA two spectra are acquired: one spectrum with control saturation, and one with a J-editing (saturation) pulse applied at approximately 1.9 ppm which modulates the phase evolution of the multiplet GABA resonance at 3 ppm (Bottomley, 1987; Mescher et al., 1998). The resonance of GABA at 3 ppm is J-coupled to the resonance of GABA at 1.9ppm. The difference formed between the regular and edited spectrum thus leaves a distinct GABA signal, whilst the signal arising from molecules unaffected by the editing pulse (e.g., creatine) is largely subtracted out. By allowing GABA to be separated from stronger overlying signals of other neurochemicals, this approach improves sensitivity to GABA signal and provides reliable in vivo measures (Mullins et al., 2014). However, as two measurements are needed for one spectrum, J-difference edited sequences are more prone to subject motion and scanner frequency drift artefacts (Edden et al., 2012). When seeking dynamic, time-resolved measures of neurochemicals using event-related fMRS, the effective reduction in the sampling rate (by a factor of two) is clearly a disadvantage. Overall, the increase in chemical sensitivity comes at the expense of significant loss of SNR. Moreover, despite using a GABA-editing pulse, both GABA and overlapping macromolecules are targeted, resulting in a ‘GABA+’ signal that remains contaminated by co-edited macromolecules (Mullins et al., 2014). At 3T or lower, signal deriving from macromolecules could comprise over 50% of GABA signals in MEGA-based editing methods (Harris et al., 2015; Rothman et al., 1993). While high field MRS helps resolve confounds from macromolecules, metabolites still overlap with macromolecules at short echo time. Alternative editing approaches, such as multiple quantum coherence filtering (MQF) and Hartman-Hahn transfer, achieve significant reduction in macromolecule contribution but coediting remains the biggest challenge for spectral editing of GABA (Choi et al., 2021).

GABA can also be measured using non-edited sequences where only one measurement is needed per spectrum (Near et al., 2013). As mentioned above, particularly at lower magnetic field strengths (3T and below), this approach is limited by the overlap between GABA peaks and other neurochemicals that have higher signal. However, at ultra-high field strengths of 7T and above, GABA can be reliably measured using unedited MRS, with spectral peaks distinguished from other neurochemicals (Finkelman et al., 2022). A reliable and reproducible acquisition pipeline when using unedited sequences, first established by Tkáč et al., involves using the FASTMAP sequence to adjust first- and second-order shim terms, suppressing the water signal using the VAPOR sequence, before applying STEAM of semi-LASER MRS acquisition sequences and analyzing data using LCModel (Tkáč et al., 2001). Direct comparison of edited and unedited measures of GABA at 7T suggests GABA/tCr ratios are similar across these two acquisition approaches (Hong et al., 2019). In the context of event-related fMRS, the benefit of using unedited sequences is that it allows for simultaneous quantification of ~20 different neurochemicals and shorter echo times, resulting in superior SNR. These features of unedited sequences may be advantageous for the application of event-related fMRS where temporal resolution and data quality are a priority.

In an effort to determine how differences between sequences affect neurochemical measurements, several studies have compared edited and non-edited sequences for static MRS, with variable results. Some studies have reported no significant differences between edited and non-edited sequences (Hong et al., 2019; Near et al., 2013; Terpstra et al., 2016). Other studies have shown an advantage to using edited sequences: certain metabolites of interest (such as glutathione) may be easier to detect with editing (Sanaei Nezhad et al., 2017) while edited GABA quantification is reported to be superior but only after removal of signal attributed to macromolecules (Chen et al., 2015). By contrast, direct comparison between edited and non-edited sequences at 7T reveals better reproducibility of glutamate and GABA quantification when using non-edited sequences, particularly those with longer echo time (TE of 80 ms versus 42 ms) (Finkelman et al., 2022). However, at present, there are no studies directly comparing edited and non-edited sequences for event-related fMRS. Nevertheless, at ultra-high field strengths (7T and above), unedited sequences may be considered preferable for event-related fMRS given the superior temporal resolution and higher SNR which together favour reproducibility (Finkelman et al., 2022). Consistent with this line of reasoning, Koolschijn et al. used an unedited semi-LASER sequence at 7T to reveal event-related changes in glutamate and GABA during memory recall, with good spectral data quality indicated by signal-to-noise ratio, linewidth and intra-subject test-retest coefficients of variation (CoV) (Koolschijn et al., 2021).

Additional measures can be taken to improve SNR of fMRS data acquisition in both humans and animal models. SNR is affected by both the size of the volume of interest (VOI) and the number of spectra per condition. However, it is worth noting that increasing VOI to improve SNR may reduce anatomical specificity. This is particularly relevant for fMRS where the target tissue is typically a small region of (cortical) grey matter, far smaller than the total voxel volume. The size of the VOI may thus be selected based on a trade-off between SNR and anatomical specificity.

When measuring static GABA at 3T, the consensus recommendation is to acquire 128 transients, from ~27 ml VOI for edited MRS and from ~3.4 ml for unedited spectra (Lin et al., 2021). Similar figures may be suitable for event-related fMRS but there are currently not enough published studies to make these estimates. The minimum number of spectra required per condition will also depend upon the number of subjects and spectral quality. Previous studies have shown minimal change in test-retest CoV for glutamate when going from 8 to 16 spectra (Terpstra et al., 2016). For studies with comparable data quality and power, subjects may therefore be included for analyses that have 8 or more spectra for any given condition (Koolschijn et al., 2021). However, given the challenges associated with obtaining robust event-related measures of neurochemicals tens or hundreds of spectra per condition is preferable. As an example, Koolschijn et al., included on average ~40 spectra per condition, across 18 participants, with average SNR of 51.1 and average CoV for glutamate and GABA equal to 2.68 and 8.74, respectively.

To further improve SNR, dielectric pads made from barium or calcium titanate can be used to create a “hot-spot” in the radio-frequency distribution in the volume of interest (Brink & Webb, 2014; Teeuwisse et al., 2012). In preclinical work, a cryoprobe may be used to increase SNR by a factor of 2 or more, in part overcoming the difficulty in animal models of acquiring MRS data from sufficiently small voxels to allow for anatomical specificity (Ligneul et al., 2021; Tkáč et al., 2004). Overall, we recommend that spectral quality is assessed during acquisition and voxel placement and shimming repeated if data quality is not considered sufficiently high.

## Analysing event-related fMRS data

Having discussed how event-related fMRS can be optimised through choice of task-design and sequence, a further consideration is the analysis pipeline. A number of software packages have been developed in recent years for processing and quantification of spectra (Clarke et al., 2021; Edden et al., 2014; Gajdošík et al., 2021; Naressi et al., 2001; Oeltzschner et al., 2020; Provencher, 1993, 2001; Reynolds et al., 2006; Stefan et al., 2009; Wilson, 2021; Wilson et al., 2011). However, despite attempts to establish agreement in the MRS community (Andronesi et al., 2020; Choi et al., 2021; Lin et al., 2021; Near et al., 2021; Öz et al., 2020; Tkáč et al., 2021), the most appropriate analysis pipeline remains the subject of ongoing debate (Jenkins et al., 2021; Marjańska et al., 2022). Here we will start by focusing our discussion on the most widely used algorithm, namely LCModel. LCModel is often considered the de facto “gold-standard” method of modelling, being used for virtually all MRS methods in the brain, accounting for around 90% of citations reported across the various available software packages (Zöllner et al., 2021).

Event-related fMRS analyses using LCModel build on analytical approaches designed to quantify the concentration of neurochemicals from spectral averages. To obtain an event-related measure, one approach involves first assigning a condition to each spectra, before averaging spectra within condition, as illustrated in Fig. 1b-c. The advantage of this approach is that it can be fully scripted and employs the most widely used algorithm that is now freely available and open source. The disadvantage is that it does not provide a means to readily account for the metabolite response function, or control for potential physiological or motion related confounds.

When using LCModel to analyse event-related MRS data, certain features should be considered. Namely, LCModel leverages prior knowledge to assume the concentration of neurochemicals that reside at relatively low concentration remain fixed within a predefined, physiological range (Marjańska et al., 2022). The predefined concentration range is defined relative to other more abundant neurochemicals. These prior (or ‘soft’, nonlinear) constraints are applied on the ratios of lipid components and macromolecule components. By default, soft constraints are also applied to a number of neurochemicals that include GABA, aspartate, glycine, scyllo and tau. For example, in LCModel the predefined range for GABA is defined as follows:

$$\sqrt{\frac{\left[GABA\right]}{\left[Big3\right]}=0.04\pm 0.04},$$

$$\sqrt{\left[Big3]=[totNAA]+[totCr]+3[totCho\right]}$$

A Gaussian prior is imposed with a mean and standard deviation 0.04 times the weighted sum of the total N-acetylaspartate (totNAA), total creatine (totCr) and total choline (totCho) concentrations which represent the approximately invariant ‘Big3’. These priors are designed to reduce biases in the overall analyses that are introduced by allowing relatively weak neurochemicals to assume values at unrealistic concentrations (Provencher, 2021). Removing these default priors may be ill advised when using MRS to obtain a static readout by averaging spectra through time. However, we propose default priors are not suitable for event-related fMRS where the measure of interest is typically the change in concentration of a given neurochemical between different conditions of interest.

**Figure 3:**
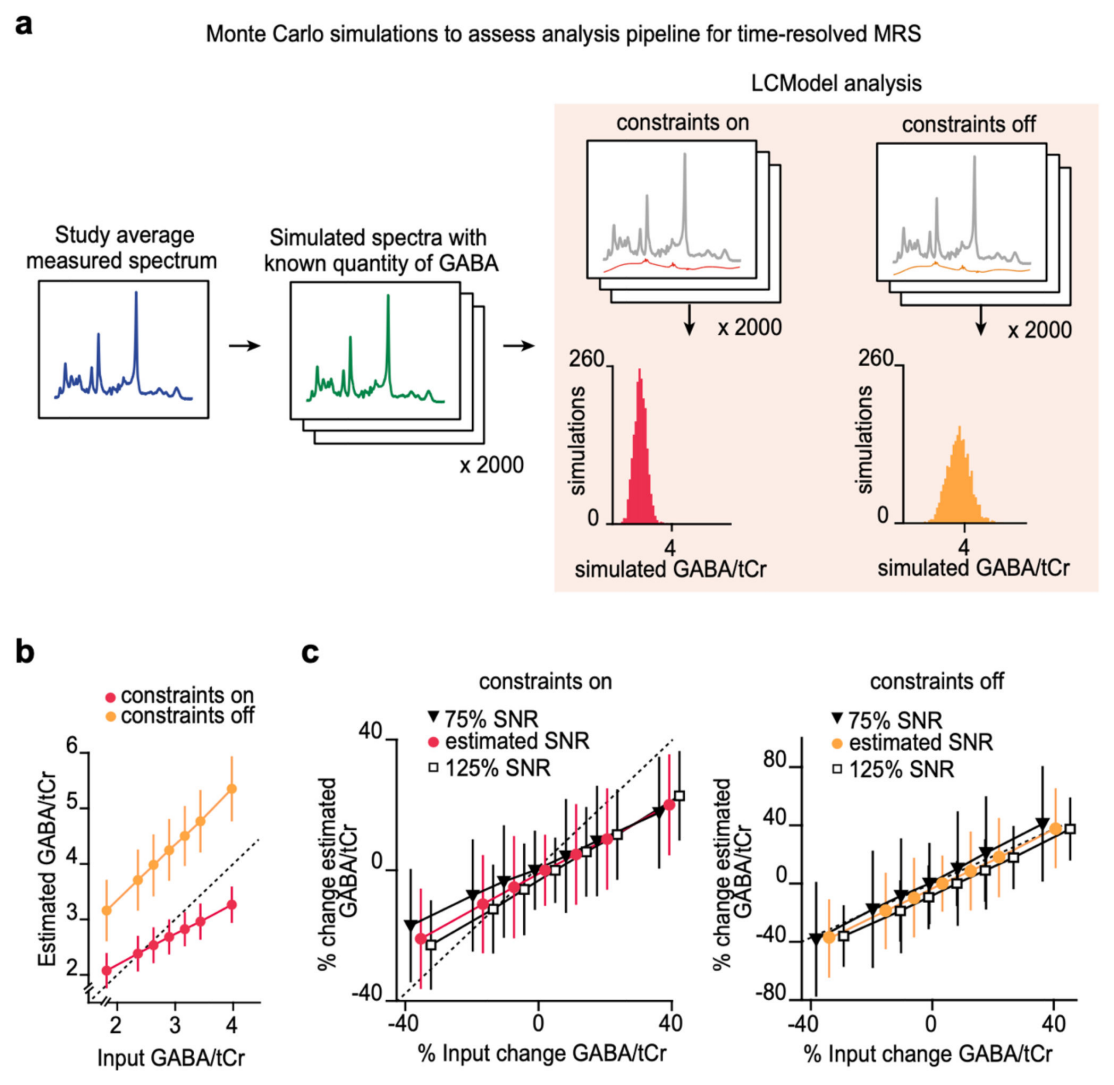
Monte Carlo simulations to assess the effect of including/removing prior constraints on GABA. **a.** Schematic of the simulation procedure. Sets of synthetic spectra were generated with changes in GABA imposed, which included multiples (±0.5, ±1, ±2) of the observed effect size in (Koolschijn et al., 2021). Model fitting was performed in LCModel with ‘constraints on’ (red) and ‘constraints off’ (orange). **b.** For different imposed changes in GABA, the mean ± standard deviation for the resulting GABA:total Creatine (tCR) concentrations (n = 2000) are shown, as estimated with LCModel using ‘constraints on’ and ‘constraints off’. The dashed line represents agreement between the imposed and estimated GABA:tCr concentration. **c.** With ‘constraint on’ the measured changes deviated from the imposed changes at both higher and lower SNRs. With ‘constraints off’, the estimated changes closely reflected the imposed changes in GABA at different SNRs. These simulations show that across a range of different SNRs, dynamic changes in GABA are robustly reflected in the measured GABA concentrations using ‘constraints off’. Adapted with permission from Koolschijn et al. (2021).

To demonstrate how prior constraints in spectral fitting algorithms can mask dynamic changes in event-related GABA, Koolschijn et al. (2021) used Monte Carlo simulations to generate sets of synthetic fMRS data while preserving the level of noise observed in a 7T unedited MRS dataset (Figure 3). In the synthetic spectra GABA was artificially added or removed before LCModel was applied to quantify neurochemicals, either with or without using prior constraints (Figure 3A). These simulations show that when using prior constraints, GABA estimates are lower and show reduced variability, yet dynamic sensitivity is reduced (Figure 3B-C). On the other hand, if prior constraints are removed, GABA estimates are higher and show more variability, but dynamic changes in GABA are more reliably detected (Figure 3B), including at different SNRs (Figure 3C). These simulations show that when using LCModel to quantify event-related changes in neurochemicals such as GABA, foregoing the use of prior constraints increases the sensitivity of the spectral fitting algorithms. Notably, rather than assessing absolute concentrations, when prior constraints are removed a difference or ratio between experimental conditions should be used to detect meaningful changes in neurochemical concentrations. More broadly, this example illustrates how Monte Carlo simulations can be used to scrutinise analysis pipelines used to detect event-related measures of neurochemicals. This approach builds on a template designed to assess quantification reliability of neurochemicals that have spectral overlap with more abundant neurochemicals, as illustrated for glutathione (Deelchand et al., 2016).

Recently, new open-source MRS analysis software libraries have been published (Clarke et al., 2021; Gajdošík et al., 2021; Oeltzschner et al., 2020; Wilson, 2021), with some integrating a modular and easily extensible design. This plethora of new analysis software could give the field an opportunity to rapidly innovate, develop and test new ideas for the field of event-related (and blocked design) fMRS. For example, software libraries such as FSL-MRS allow for implementation of a General Linear Model (GLM) that can readily account for the metabolite response functions and include ‘nuisance regressors’ to control for potential confounding factors (Clarke et al., 2021). This approach may be considered analogous to fMRI data analyses where GLMs are widely used to assess dynamic changes in the BOLD signal during event-related tasks.

As an example, Ligneul et al. recently used a GLM to analyse fMRS data by assessing the time courses of different metabolites in response to blocks with 24 s periods of visual stimulation in anaesthetised mice. In this study, consecutive spectra in time were first summed across 8 repetitions to provide a moving average with an effective resolution of 12 s. A GLM was then applied to the resulting time course of different neurochemicals, to compare potential alternative models as explanatory variables. The authors report that while NAA varied significantly with the individual 24 s periods of stimulation, glutamate varied more slowly, showing increased signal across the entire ‘active’ period of stimulation relative to a subsequent ‘recovery’ period without stimulation. Ligneul et al. were also able to estimate the metabolite response function for different metabolites using a finite impulse response approach. When applied to the moving average for each metabolite, with effective temporal resolution of 12 s, the response function for glutamate ramped up for ~3 minutes, only decreasing at the end of the blocked stimulation. However, the blocked design and relatively low temporal resolution of 12 s may have precluded insight into more rapid dynamics. Indeed, compared to the HRF, the glutamate response function is hypothesised to peak much earlier, at around 500 ms after stimulus onset, returning to baseline before the peak in the HRF which typically occurs around 5 s after stimulus onset (Mullins, 2018) To improve sensitivity of GLM-based analyses for detecting event-related changes in neurochemicals there is critical need for an event-related fMRS study dedicated to mapping metabolite response functions.

A key advantage of a GLM-based approach is that ‘nuisance regressors’ can be included in the design matrix to control for potential confounds. These nuisance regressors may include: (1) movement parameters, including involuntary motion; (2) physiological parameters, such as harmonics of the cardiac spectrum; (3) hardware related drift due to warming or cooling effects; (4) confounds that derive from BOLD artefacts. BOLD related artefacts involve linewidth narrowing in the MRS spectra, due to increases in T2/T2* that accompany increases in BOLD (Boillat et al., 2020; Zhu & Chen, 2001). If left uncorrected, spectral line narrowing due to the BOLD signal can bias fMRS metabolite quantification, giving an apparent increase in metabolite concentration (Bednařík et al., 2015; Mangia et al., 2006). To correct for BOLD-related line narrowing, several different approaches can be taken.

One approach involves estimating narrowing of the total creatine singlet peak (tCr) at 3.03 ppm (Ip et al., 2017; Mangia et al., 2007; Zhu & Chen, 2001). Under the assumption that the total creatine singlet peak remains stable during brain activation, changes in the tCr linewidth may be attributed to the BOLD signal. A second approach involves using changes in linewidth of the water peak to estimate BOLD-related line narrowing (Ligneul et al., 2021), under the assumption that BOLD effects impart similar dynamics on both water and metabolite signals.

Corrections for BOLD-related artefacts may also be applied directly to fMRS data. For example, Ip et al., 2017, simultaneously acquired fMRI and fMRS data from primary visual cortex using a blocked design. Compared to baseline they measured a 2% narrowing of the tCr line widths during stimulation which correlated with changes in BOLD (Ip et al., 2017). Using the tCr line width narrowing, subject specific corrections were applied to the MRS spectra during periods of stimulation, revealing a significant change in corrected glutamate but not GABA during stimulation. Similarly, Ligneul et al. 2021, used the water peak to estimate BOLD effects in the MRS spectra before applying line broadening to the timecourse of all metabolites during predefined BOLD events, demonstrating significant increases in corrected glutamate during blocks of visual stimulation (Ligneul et al., 2021). Data acquired using event-related fMRS designs may similarly be corrected for line narrowing due to the BOLD signal and using a GLM for this purpose may be particularly effective.

Regardless of the particular analysis pipeline implemented, data quality remains critical for reliable event-related fMRS. To avoid sacrificing SNR at the expense of improved temporal resolution, several approaches can be taken, which include averaging sufficient number of spectra per task condition (Koolschijn et al., 2021), using a sliding window to obtain a time course (Ligneul et al., 2021), and/or averaging data across sufficient number of participants to obtain group-level spectra (Apšvalka et al., 2015). We further recommend implementing a process of data filtering to discard data that are not of sufficiently high data quality. Depending on the acquisition protocol implemented, a conservative rule of thumb could involve discarding any spectra where the water residual signal is greater in amplitude than the NAA or total creatine peak.

As with other forms of MRS data, data quality metrics can be applied to event-related fMRS data. These include, SNR, linewidth, frequency offset, Cramer-Rao Lower Bounds (CRLBs), and inter- or intra-subject coefficients of variation (CoV). While benchmark values of these parameters have not yet been defined for event-related fMRS, cross-site MRS studies have reported these parameters for GABA (Mikkelsen et al., 2017) and individual studies have reported values in the context of event-related fMRS (Koolschijn et al., 2021).

We also note that event-related MRS is highly sensitive to biases of spectral fitting which may arise due to confounding differences between conditions. For example, differences in the number of spectra between condition will result in differences in SNR that could introduce apparent changes in metabolite concentration between condition. As illustrated by Koolschijn et al., such biases can be faithfully captured using simulations and permutation testing (Koolschijn et al., 2021), methods that provide a means to assess the change in metabolite concentration that would be expected by chance.

## Reproducibility of event-related fMRS

The above discussion on measuring and analysing event-related fMRS data may provide researchers with guidelines for best practice. Nevertheless, reproducibility remains a concern for event-related fMRS studies, and fMRS more generally. Indeed, recent meta-analyses of fMRS studies report significant heterogeneity across different studies (Pasanta et al., 2023), which may be attributed to variation in task design including stimulus type, brain region, sequence parameters, scanner field strength, analysis parameters, and choice of software. For example, Mullins et al. reported strong evidence to suggest glutamate in ACC correlates strongly with the subjective level of pain experienced by participants (Mullins et al., 2005). By contrast, Archibold et al. found an increase in ACC glutamate only at the onset of a painful stimulus, with no evidence to suggest glutamate tracks subsequent reports of pain (Archibald et al., 2020). To facilitate reproducibility, several groups have recently published consensus recommendations for best practice. For example, *MRSinMRS* was established from a consensus group of MRS experts to provide minimum guidelines for the reporting of MRS methods and results, including the standardized description of MRS hardware, data acquisition, analysis, and quality assessment (Lin et al., 2021). Similarly, *MRS-Q* was developed from existing consensus to allow for the assessment of MRS quality (Peek et al., 2020), and Choi et al. describe consensus recommendations for using editing sequences (Choi et al., 2021). Although these consensus papers were originally proposed for static MRS, they may be applied and adapted to fMRS studies, including those using event-related designs (Pasanta et al., 2023).

## Interpreting event-related fMRS data

While event-related fMRS studies show convincing evidence for rapid changes in neurochemicals, the origin of the underlying signal remains controversial. As briefly mentioned above, neurochemicals are present in multiple cellular compartments that include the cytoplasm, extracellular space and vesicular pools. Although the spatial resolution of MRS is insufficient to distinguish between these different neurochemical pools, there is evidence to suggest variation in neurochemical visibility to MRS across different compartments (Kauppinen et al., 1994a; Kauppinen & Williams, 1991; Yüksel & Öngür, 2010). Theoretically, MRS is unlikely to be sensitive to changes in the extracellular pools of glutamate and GABA which accompany neurotransmitter release as the concentration of glutamate and GABA in the extracellular pools is around 100-fold smaller than in the intracellular pool (Myers et al., 2016). In addition, post-mortem studies suggest MRS is not sensitive to intracellular pools that reside in the mitochondria or vesicles (De Graaf & Bovée, 1990; Kauppinen & Williams, 1991). Instead, some studies suggest MRS is considered more sensitive to unconstrained intracellular metabolic pools which reside at sufficiently high concentration in the neuronal cytoplasm (Najac et al., 2014; Rae, 2014). However, given these studies mostly focused on neurochemicals that reside at relatively high concentration, and some were performed at low field strengths (3T and below), it remains unclear whether findings can be extrapolated to other neurochemicals, including GABA, particularly when measured at higher field strength.

Task-induced changes in MRS measures may be explained by tight coupling between intracellular metabolic pools of glutamate and GABA and neurotransmitter release. A tight coupling between metabolic and neurotransmitter pools can be observed at rest, where a 1:1 relationship is reported between the rate of glutamine-glutamate cycling and neuronal oxidative glucose consumption (Rothman et al., 2003; Shen et al., 1999; Sibson et al., 1998). During sensory stimulation a transient uncoupling has been observed with a short-lived mismatch between glucose utilization and oxygen consumption (Fox et al., 1988; Fox & Raichle, 1986), particularly during stimulation protocols that alternate between high intensity and quiescent periods (Gjedde et al., 2002). This short-lived uncoupling is thought to be selective to intense and transient sensory stimulation, and is not observed during anaesthesia or during certain stimulation protocols. This short-lived uncoupling may provide a basis for task-induced changes in MRS measures of glutamate and GABA that reflect functionally relevant activity. In the case of glutamate, the short-lived uncoupling between oxidative metabolism and glutamate-glutamine cycling would lead to an increase in synthesis of glutamate relative to degradation. Opposing dynamic changes in glutamate and GABA may therefore reflect a transient recalibration or shift in the balance between excitation and inhibition (E/I) at the physiological level.

Consistent with this line of reasoning, a recent study in mice used a tactile stimulation paradigm to compare data acquired using two-photon microscopy and data acquired using a blocked-design with fMRS (Takado et al., 2022). In the awake state, Takado et al. found that changes in MRS-measured Glu and GABA concentrations were overall in accordance with the changes in excitatory and inhibitory neural activities, respectively. These findings suggest that functional changes in glutamate and GABA measured using a blocked design with fMRS are reflective of changes in neurotransmission.

However, although metabolic processes that allow for net changes in the concentration of glutamate and GABA may account for changes in neurochemicals reported using blocked designs, they are considered too slow to account for more rapid fluctuations detected using event-related MRS. Moreover, the average change in neurochemical concentration reported using event-related designs (~14%) (Mullins, 2018), is considered too high to be accounted for by known synthesis or degradation metabolic pathways. An alternative possibility is that event-related MRS is sensitive to rapid changes in the concentration of metabolites between different cellular compartments that accompany neural activity. In total it is estimated that due to differences in T2 relaxation between bound and unbound neurochemicals, up to 30% of glutamate is invisible to MRS at any one time point (Kauppinen et al., 1994b; Kauppinen & Williams, 1991; Pirttilä et al., 1993). Therefore, with neural activity, the shift in glutamate from pre-synaptic vesicles to more visible synaptic, extracellular and astrocytic pools may plausibly account for increases in event-related fMRS signal (Jelen et al., 2018). In addition, the rate of vesicle release and refilling are not equivalent, with in vitro evidence demonstrating that refilling is slower than release (Stevens & Tsujimoto, 1995). The *compartmental shift hypothesis* thus proposes that changes in visibility of neurochemicals can occur without the need for an actual change in the overall ‘total’ concentration of neurochemicals (Mullins, 2018).

To further establish the origin of event-related fMRS signal, one approach involves using computational modelling. Lea-Carnall et al. modelled neurotransmitter dynamics at the level of the cellular compartments where glutamate and GABA cycle between packaged vesicles, the synaptic cleft and recycling/repackaging in the astrocytic and/or neuronal cytosol. Simulations of neurochemical cycling were then combined with a macroscopic model to predict MRS-derived signal using a mean-field reduction of activity across a large ensemble of neurons. As predicted by the compartmental shift hypothesis, changes in extracellular and cytosolic pools in the model could be used to explain event-related fluctuations in neurochemicals. Therefore, increases in MRS-quantified glutamate occur when glutamate shifts from the vesicular compartment to the cytosolic compartments, while a decrease in MRS-quantified GABA occur when GABA shifts in the reverse direction from cytosolic compartments into the vesicular compartment (Lea-Carnall et al., 2023). Importantly, the modelling shows that a new steady-state is reached within 5 seconds in response to a change in activity level. Thus, these findings suggest that compartmental shifts in glutamate and GABA can, in theory, account for changes in neurochemical reported within a few seconds from stimulus onset (Lea-Carnall et al., 2023), as reported using event-related fMRS. Interestingly, the modelling aligns with empirical results in predicting larger changes in event-related compared to blocked designs, with increased sensitivity to compartmental shifts observed when using longer echo times.

To establish empirical evidence in support of the compartmental shift hypothesis, we need to better understand how MRS visibility changes as neurotransmitters move between different cellular compartments. Two important directions for future fMRS research are to first establish the complex relationship between fMRS-derived neurochemical measures and compartmental shifts in glutamate and GABA; and second, establish the relationship between different compartments of glutamate and GABA and physiological measures such as neurotransmitter release and neuronal spiking activity. Moreover, fMRS research needs to identify the effect of different sequence parameters, such as echo time, on these relationships. Diffusion-weighted fMRS, a new method that can distinguish neurochemicals in different cellular compartments (Branzoli et al., 2013; Cao & Wu, 2017; Palombo et al., 2017; Shemesh et al., 2017), may provide important insight. Diffusivity varies between cellular compartments, with low diffusivity in the spheres correlating with high mitochondrial viscosity, and higher diffusivity in the synaptic cleft (Vincent et al., 2021). In addition to computational modelling, diffusion-weighted imaging may therefore help reveal the origins of signal reported using event-related fMRS paradigms.

## Concluding remarks

fMRS in combination with event-related task designs has been successfully used to measure changes in neurochemical concentration at relatively high temporal resolution across a number of different brain regions. While more work is needed to appropriately interpret these rapid changes in neurochemicals, the reported functional changes detected using event-related fMRS are relatively large compared with those reported using blocked designs. In this commentary, we provide guidance and advice to those researchers interested in implementing event-related fMRS. We show how event-related fMRS can provide insight into computations underpinning cognition, particularly when careful consideration is given to the task-design, MRS sequence and analysis pipeline. Furthermore, we illustrate how event-related fMRS can be successfully combined with other modalities, including electrophysiology (Lally et al., 2014) and fMRI BOLD (Apšvalka et al., 2015), even in an interleaved manner (Koolschijn et al., 2021). By capturing dynamic, task-relevant changes in neurochemicals, event-related fMRS therefore promises to be a valuable tool that can complement alternative non-invasive methods. To conclude, we propose that event-related fMRS provides an opportunity to test hypotheses guided by computational and cognitive neuroscience, on a timescale relevant for understanding the neural basis of human cognition and behaviour.

## Figures and Tables

**Table 1:** Summary of findings reported from event-related fMRS studies

Study	Metabolites of interest	Field strength	Sequence type	ROI	Stimulus duration	Cognitive process	Sample size	Effect reported
Mangia et al., 2003	Lactate	1.5 T	PRESS	V1	Time- locked 1 s	Visual stimulation	5	Lac ↓40%
Nishitani, 2003	Creatine/Phosph ocreatine, Choline	3 T	PRESS	Hippocampus	Stimulus- locked, 1.8-2.2 s	Attention	10	Cho ↑75%*,** Cr ↑60%*,**
Lindner et al., 2017	Choline	3T	PRESS	Parietal- Occipital cortex	2.5 s	Attention	16	Cho↑61%*
Gussew et al., 2010	Glutamate	3T	PRESS	Insular cortex	5 s	Pain	6	Glu ↑18%
Lally et al., 2014	Glutamate	3T	PRESS	Lateral occipital complex	Time- locked, 700 ms	Visual perception (abstract vs objects)	13	Glu ↑11%
Apšvalka et al., 2015	Glutamate	3T	PRESS interleaved with BOLD	Lateral occipital complex	Stimulus- locked 700 ms	Repetition suppression	13	Glu ↓13%
Cleve et al., 2015	Glx and GABA+	3T	MEGA- PRESS	Occipital cortex, anterior cingulate	Stimulus- locked 1 s	Pain	8-15	Glx ↑15.7%, 21.5%, GABA+ ↓12.7%, 15.1%
Koolschijn et al., 2021	Glutamate and GABA	7T	semiLASER interleaved with BOLD	V1	2 s	Memory	19	GABA ↓6.8%

* estimated from figures

** difference between right and left hippocampus, relative to resting condition
